# Effects of Human Adipose Tissue-Derived and Umbilical Cord Tissue-Derived Mesenchymal Stem Cells in a Dextran Sulfate Sodium-Induced Mouse Model

**DOI:** 10.1089/biores.2019.0022

**Published:** 2019-11-11

**Authors:** Shunzo Ikarashi, Atsunori Tsuchiya, Yuzo Kawata, Yuichi Kojima, Takayuki Watanabe, Suguru Takeuchi, Katsuhide Igarashi, Maky Ideta-Otsuka, Katsuyuki Oki, Masaaki Takamura, Shuji Terai

**Affiliations:** ^1^ Division of Gastroenterology and Hepatology, Graduate School of Medical and Dental Sciences, Niigata University, Niigata, Japan.; ^2^ Laboratory of Biofunctional Science, Hoshi University School of Pharmacy and Pharmaceutical Sciences, Tokyo, Japan.; ^3^ Life Science Tokyo Advanced Research Center (L-StaR), Hoshi University School of Pharmacy and Pharmaceutical Sciences, Tokyo, Japan.; ^4^ BioMimetics Sympathies, Inc., Tokyo, Japan.

**Keywords:** adipose-derived mesenchymal stem cells, colitis, dextran sulfate sodium, inflammation, intestinal flora, tumor necrosis factor, umbilical cord-derived mesenchymal stem cells

## Abstract

Mesenchymal stem cells (MSCs) can be acquired from medical waste. MSCs are easily expanded and have multiple functions, including anti-inflammatory effects. We evaluated the effects of human adipose tissue-derived MSCs (AD-MSCs) and umbilical cord tissue-derived MSCs (UC-MSCs) in a dextran sulfate sodium (DSS)-induced mouse model. Human AD-MSCs and UC-MSCs (1 × 10^6^ cells) were injected intravenously into a 7-day DSS-induced colitis model. The therapeutic effects of cell origin, injection timing, and supernatants obtained from MSC cultures were evaluated. We also analyzed messenger RNA (mRNA) expression in MSCs, tissues, and intestinal flora. AD-MSCs and UC-MSCs were found to show strong anti-inflammatory effects when injected on day 3 in a mouse model. On day 11, the mRNA levels of inflammatory factors in colon tissues were significantly decreased after injection of MSCs on day 3. Supernatants from MSCs culture decreased mRNA levels of tumor necrosis factor (*Tnf*)-α, but had reduced therapeutic effects compared with MSC cell injection. RNA sequencing using colon tissues obtained the day after cell injection revealed changes in the TNF-α/nuclear factor-κB and T cell receptor signaling pathways. Additional analyses showed that several factors, including chromosome 10 open reading frame 54, stanniocalcin-1, and TNF receptor superfamily member 11b were increased in MSCs after adding serum from DSS colitis mice. Furthermore, both AD-MSCs and UC-MSCs maintained the balance of intestinal flora. In conclusion, AD-MSCs and UC-MSCs showed therapeutic effects against inflammation after early cell injection while maintaining the intestinal flora. Although supernatants showed therapeutic effects, cell injection was more effective against inflammation.

## Introduction

Inflammatory bowel diseases (IBDs) comprise chronic inflammatory disorders and include ulcerative colitis (UC) and Crohn's disease (CD). The pathogenesis of IBDs is highly complex and involves abnormal immune reactions caused by environmental factors, intestinal antigens and bacteria, and diet; however, the underlying mechanisms are still not completely understood.^1,2^ The mechanisms of inflammation have been extensively analyzed, and multiple potential therapeutic targets have been identified. Therapy is focused on control of inflammation and mucosal healing rather than complete cure of the disease.

The outcomes of IBD treatment are dramatically improved by immunomodulators and molecular targeted therapies, particularly antitumor necrosis factor (TNF)-α antibodies. Anti-TNF-α antibodies can affect a wide range of cytokines, chemokines, and related molecules and are thus highly effective for reducing inflammation. However, the availability of these drugs is limited, and more specific targets are needed.^3^

Ustekinumab, which targets interleukin (IL)-12/23p40, has been used in the clinical setting. Risankizumab, which targets IL-23, is currently being evaluated in clinical trials. Janus kinase inhibitors, which target various intracellular signaling pathways involved in cytokine production, and antiadhesion molecules, which target α4β7 integrin to prevent lymphocyte recruitment to the gut, have also been evaluated in the clinic. In addition, many new targets are being developed and assessed in clinical trials.

Although recent developments in medical treatments have resulted in improved treatment outcomes, in some patients, these therapies are ineffective, or the effects of therapy may be insufficient. Moreover, drugs are often associated with various side effects, including non-Hodgkin's lymphoma and hepatosplenic T cell lymphoma.^4–6^ Uncontrolled inflammation in patients can also lead to various complications, necessitating additional surgeries or procedures and therefore decreasing the quality of life. For example, if intestinal inflammation cannot be effectively controlled, UC is associated with increased risk of dysplasia and cancer. Furthermore, costs of these therapies are high. Therefore, there is a need to develop and explore new therapeutic drugs and approaches.

Mesenchymal stem cells (MSCs) have been shown to have applications in the treatment of IBDs. They can differentiate into osteoblasts, chondrocytes, and adipocytes and can be obtained not only from bone marrow but also from medical wastes, such as adipose tissue, umbilical cord tissue, and dental pulp.^7,8^ MSCs must express CD105, CD73, and CD90 and lack expression of CD45, CD34, CD14 or CD11b, CD79a or CD19, and human leukocyte antigen (HLA) type DR surface molecules.^9^ Moreover, MSCs have been reported to have multiple effects, including anti-inflammatory, immunoregulatory, antioxidative, angiogenic, and antifibrotic effects. They express HLA class I molecules at low levels, but do not express HLA class II antigens or CD80, CD86, CD40, or CD40L costimulatory molecules.^10,11^ MSCs show low antigenicity, expand relatively easily, and produce factors such as cytokines, chemokines, growth factors, and exosomes, thereby supporting their applications as an autologous and allogeneic cell source in regenerative medicine.^12^

According to ClinicalTrials.gov, 26 clinical trials using MSCs for IBDs are registered; most of these studies are evaluating the use of MSCs in CD. In CD, MSC therapy for anal fistula by direct injection of MSCs into the fistula has been frequently reported. However, MSCs have not been extensively studied in UC or luminal CD. The cell dose, cell injection frequency, cell origin, and administration route differ in the studies using MSCs, and the optimal approach is often not clear.^8^

Accordingly, in this study, we investigated the inflammation reduction efficacy of human adipose tissue-derived MSCs (AD-MSCs) and human umbilical cord tissue-derived MSCs (UC-MSCs) using a dextran sulfate sodium (DSS)-induced mouse model of colorectal inflammation, a representative mouse colitis model.

## Materials and Methods

### Mice

Ten- to twelve-week-old C57BL/6 male mice were purchased from Charles River Laboratories International, Inc. (Yokohama, Japan). They were housed in a specific pathogen-free environment and kept under standard conditions with a 12-h day/night cycle and access to food and water *ad libitum*. All animal experiments were conducted in compliance with regulations and approved by the Institutional Animal Care and Committee at the Niigata University.

### MSC preparation

Human abdomen or buttock adipose tissues were collected with informed consent from patients receiving regenerative medicine using AD-MSCs at SunfieldClinic (Tokyo, Japan). Human umbilical cord tissues were collected from full-term pregnant women who provided informed consent at Narita Ladies' Clinic (Saitama, Japan). AD-MSCs and UC-MSCs were isolated from samples, and stromal vascular fraction were cultured with sf-DOT (BioMimetics Sympathies, Inc., Tokyo, Japan), a serum-free culture medium, at 37°C in an atmosphere containing 5% CO_2_. After reaching confluence, adherent cells were trypsinized using TrypLe Select (Thermo Fisher Scientific, Tokyo, Japan) and replated. Cells were passaged using sf-DOT three or four times, and the characteristics of AD-MSCs and UC-MSCs were verified by analysis of the differentiation, proliferation, and immunologic phenotypes. These expanded cells were a kind gift from BioMimetics Sympathies, Inc. The cells were frozen and delivered to Niigata University. After thawing, the cells were immediately washed, counted, suspended in phosphate-buffered saline (PBS), and administered to mice.

### Induction of experimental colitis and study design

**FIG. 1.**
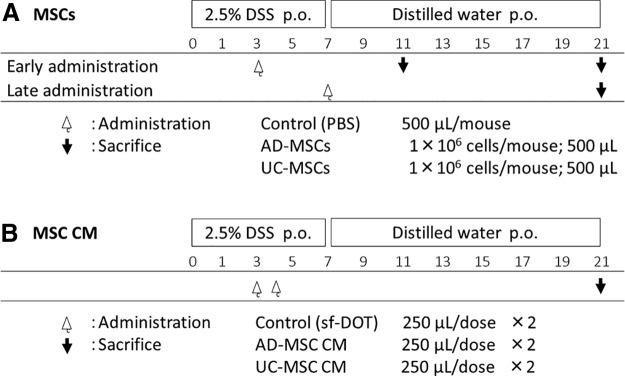
Experimental design. Colitis was induced by administration of 2.5% DSS in the drinking water *ad libitum* for 7 days. **(A)** Early administration group, mice were injected intravenously with 1 × 10^6^ human AD-MSCs, 1 × 10^6^ human UC-MSCs, or PBS on day 3 and were sacrificed on day 11 or 21; late administration group, mice were injected intravenously with 1 × 10^6^ AD-MSCs, 1 × 10^6^ UC-MSCs, or PBS on day 7 and were sacrificed on day 21. **(B)** Mice were injected intravenously with 250 μL AD-MSC CM, 250 μL UC-MSC CM, or 250 μL sf-DOT (medium for culture of AD-MSCs and UC-MSCs) alone on days 3 and 4 and were sacrificed on day 21. AD-MSCs, adipose tissue-derived mesenchymal stem cells; CM, conditioned medium; DSS, dextran sulfate sodium; PBS, phosphate-buffered saline; UC-MSCs, umbilical cord tissue-derived mesenchymal stem cells.

Colitis was induced through the administration of 2.5% DSS (molecular weight 36,000–50,000; MP Biomedicals, Santa Ana, CA) via the drinking water *ad libitum* for 7 days. Mice were divided into three groups as follows (Fig. 1): (1) injected intravenously with 1 × 10^6^ AD-MSCs, 1 × 10^6^ UC-MSCs, or PBS on day 3 and sacrificed on day 11 after the start of the experiment (early administration group [day 3 injection, day 11 sacrifice]); (2) injected intravenously with 1 × 10^6^ AD-MSCs, 1 × 10^6^ UC-MSCs, or PBS on day 3 and sacrificed on day 21 after the start of the experiment (early administration group [day 3 injection, day 21 sacrifice]); and (3) injected intravenously with 1 × 10^6^ AD-MSCs, 1 × 10^6^ UC-MSCs, or PBS on day 7 and sacrificed on day 21 after the start of the experiment (late administration group). MSCs were injected after thawing, without culture, on day 3 (early point) or day 7 (late point) because we detected weight loss and bloody stool on day 3, indicating induction of colitis; in addition, the disease activity index (DAI) was highest on day 7.

Moreover, we analyzed the therapeutic effects of MSC conditioned medium (CM). The CM of AD-MSCs and UC-MSCs was obtained by collecting culture supernatants at P3 or P4 and filtering the supernatant using a 0.22-μm filter (Cat. No. SCGPU05RE; Merck Millipore, Darmstadt, Germany). As a control, sf-DOT provided by BioMimetics Sympathies, Inc. was used. Mice were injected intravenously with 250 μL AD-MSC CM, UC-MSC CM, or sf-DOT alone on days 3 and 4 and were sacrificed on day 21.

### Evaluation of therapeutic effects

To evaluate the therapeutic effects of MSCs and MSC CM, the DAI, colon length, and histological score were analyzed. DAI was calculated by the combined scores of weight loss, stool consistency, and bleeding, as described previously.^13^ Colon lengths were measured from the anus to the cecum soon after harvesting the colon. Samples were measured as an indirect assessment of inflammation. Histological score was calculated as follows. The colon was excised, fixed in 10% formalin, embedded in paraffin wax, and sliced into 4-μm-thick sections. After hematoxylin and eosin (H&E) staining, histological evaluation was performed in a blinded manner according to a previously published scoring system.^14^ In brief, the total colitis score was determined as the sum of the three subscores (inflammation severity: 0–3 points, inflammation extent: 0–3 points, and crypt damage: 0–4 points), which were multiplied by the degree of inflammation involvement as follows: × 1, 1–25%; × 2, 26–50%; × 3, 51–75%; × 4, 76–100%. Specimens with high scores were shown to have severe histological damage. We evaluated the histological score in the medial colon because it was an appropriate location; inflammation in the distal colon was too severe, and inflammation in the proximal colon was too mild.

### Real-time polymerase chain reaction

Total RNA was reverse transcribed using a QuantiTect Reverse Transcription kit (Qiagen, Hilden, Germany). Gene expression analysis was performed using prevalidated QuantiTect primers (Supplementary Table S1) with QuantiTect SYBR reagent (Qiagen). Real-time polymerase chain reaction (PCR) was conducted using a Step One Plus Real-time PCR System (Applied Biosystems, Foster City, CA). Results were obtained using at least three separate samples, and *Gapdh* was used as the housekeeping gene. Fold change in relative gene expression, compared with that of the control, was calculated using the ΔΔCT method with pooled control samples as the calibrator.

### Next-generation sequencing

Mice with DSS-induced colitis as described above were injected intravenously with 1 × 10^6^ AD-MSCs, 1 × 10^6^ UC-MSCs, or PBS on day 3 and were sacrificed on day 4 after the start of the experiment. The excised medial colons were stored frozen at −80°C. Total RNA was extracted from these samples using an RNeasy Mini kit (Qiagen N.V., Venlo, the Netherlands) according to the manufacturer's instructions. Tissue disruption and cell lysis were performed in buffer RLT in a GentleMACS Dissociator (Miltenyi Biotec K.K., Tokyo, Japan) using the preset program (RNA_02) for total RNA extraction. Samples with an RNA integrity number of <7 were excluded from further processing and analysis.

Next-generation sequencing (NGS) was performed by Life Science Tokyo Advanced Research Center (L-StaR), Pharmacy and Pharmaceutical Science, Hoshi University. Libraries for RNA-sequencing (RNA-seq) were constructed using a SMARTer Stranded Total RNA-Seq Kit-Pico Input Mammalian (Takara Bio, Shiga, Japan) according to the manufacturer's instructions. The average library size was calculated from the obtained library DNA using a TapeStation 2200 (Agilent Technologies, Santa Clara, CA). All libraries were sequenced on a HiSeq2500 (Illumina, Inc., San Diego, CA) with single-end reads (50 bp). The 50-bp-long single-end sequence reads were mapped to a mouse genome reference sequence (mouse GRCm38/mm10), and the mapped data were imported to Strand NGS analysis software (Agilent Technologies) and used for downstream gene expression analysis.

### Analysis of intestinal flora

Mice with DSS-induced colitis, as described above, were injected intravenously with 1 × 10^6^ AD-MSCs, 1 × 10^6^ UC-MSCs, or PBS on day 3 and sacrificed on day 11 after the start of the experiment. For collection of colon content, the cecum was cut open longitudinally, and the contents were removed for analysis. Analysis of intestinal flora was performed by MIYARISAN Pharmaceutical Co. (Tokyo, Japan).

### Cap analysis of gene expression

We analyzed the characteristics of AD-MSCs and UC-MSCs in simple culture and after addition of serum to mimic the environment after administration. AD-MSCs and UC-MSCs were cultured for 2 weeks with medium changed twice weekly. Serum was obtained from mice who received DSS for 7 days. AD-MSCs and UC-MSCs were cultured with serum (final concentration: 10%) from DSS-induced colitis model mice and compared with AD-MSCs and UC-MSC treated with serum (final concentration, 10%) from normal mice. After 48 h, cells were harvested, and messenger RNA (mRNA) expression levels were analyzed by cap analysis of gene expression (CAGE) at DANAFORM (Yokohama, Japan).

### Statistical analyses

Statistical analyses were performed using GraphPad Prism6J software (GraphPad Software, Inc., La Jolla, CA) and Microsoft Excel (Microsoft Corporation, Redmond, WA). Data are presented as the means ± standard deviations. Results were assessed using Mann–Whitney U tests and Student's *t-*tests. Differences were considered significant when the *p* value was <0.05.

### Ethical considerations

All animal and human experiments were conducted in full compliance with the regulations of and were approved by the Institutional Animal Care and Committee at the Niigata University (approval No. 28-13-7 for animal studies; approval No. 2015-2486 for human studies).

## Results

### Early timing of AD-MSC and UC-MSC injection ameliorated DSS-induced colitis

**FIG. 2.**
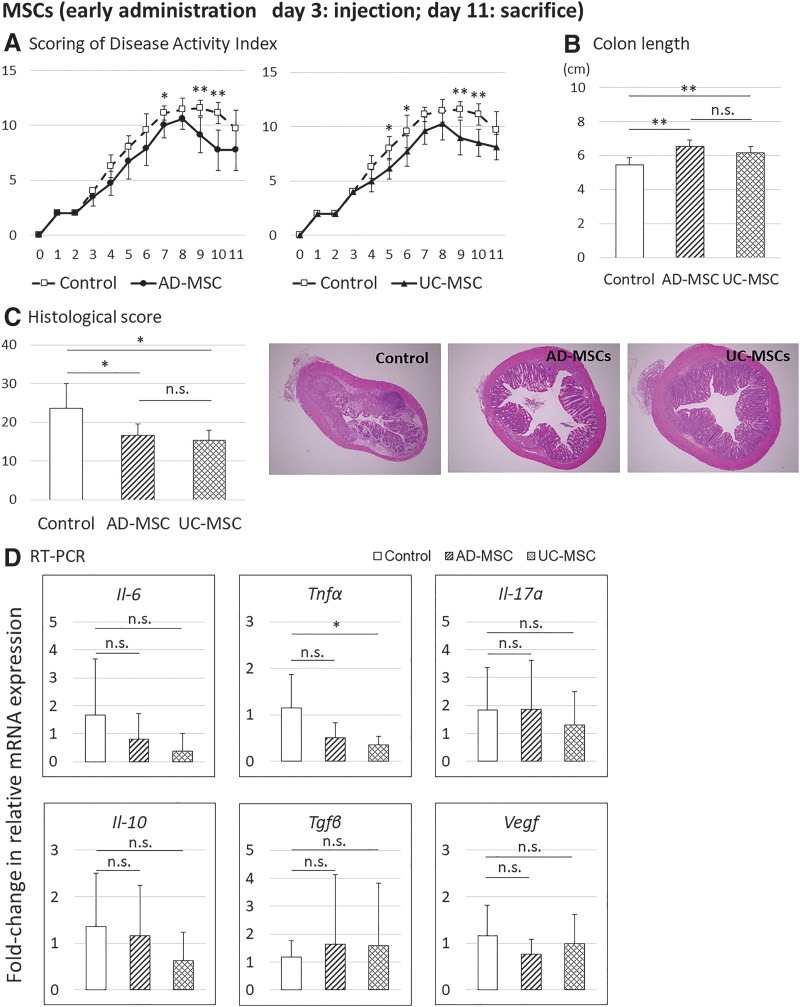
Evaluation of the therapeutic effects of AD-MSCs and UC-MSCs in a mouse model of DSS-induced colitis after administration on day 3 after initiation and sacrifice on day 11. Data are presented as the means ± SDs (*n* = 23 mice: control, 7 mice; AD-MSCs, 8 mice; UC-MSCs, 8 mice) for each experiment. **(A)** DAI scores. **(B)** Colon length. **(C)** Histological scores. **(D)** Analysis of changes in mRNA expression compared with controls, as assessed by real-time PCR, in the middle colon. *p < 0.05; **p < 0.01. DAI, disease activity index; mRNA, messenger RNA; PCR, polymerase chain reaction; SDs, standard deviations.

**FIG. 3.**
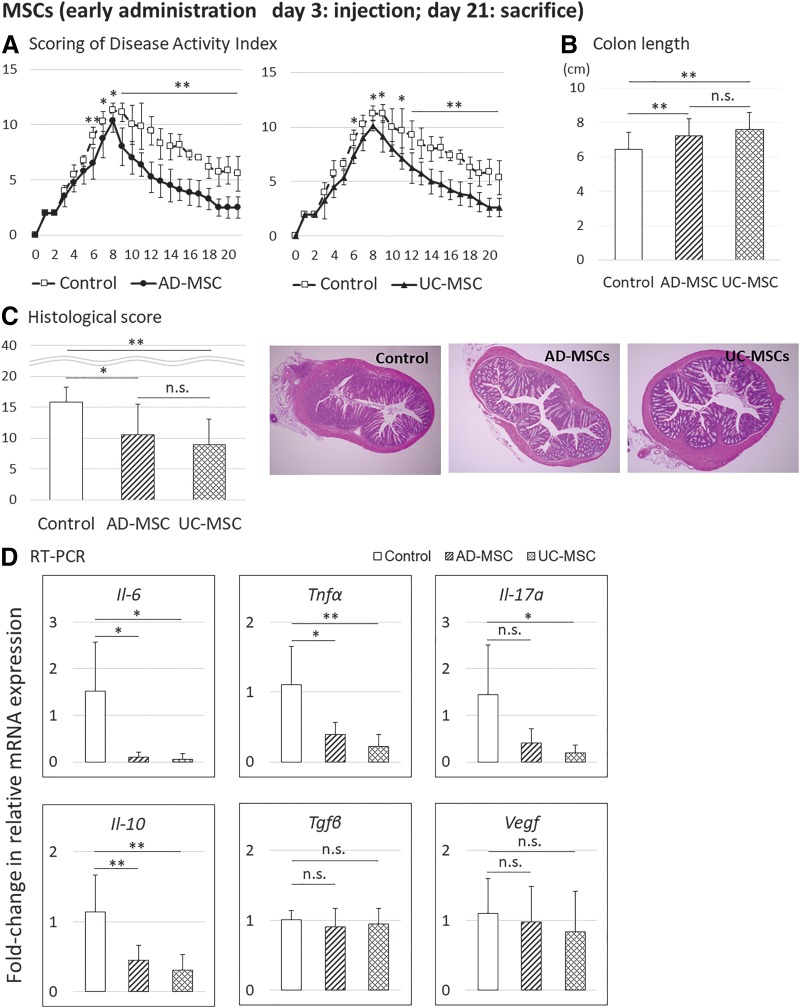
Evaluation of the therapeutic effects of AD-MSCs and UC-MSCs in a mouse model of DSS-induced colitis, after administration on day 3 and sacrifice on day 21. Data are presented as means ± SDs (*n* = 23 mice: control, 7 mice; AD-MSCs, 8 mice; UC-MSCs, 8 mice) for each experiment. **(A)** DAI scores. **(B)** Colon length. **(C)** Histological scores. **(D)** Analysis of changes in mRNA expression compared with controls, as assessed by real-time PCR, in the middle colon. *p < 0.05; **p < 0.01.

To confirm the therapeutic effects of human AD-MSCs and UC-MSCs in DSS-induced colitis and determine the appropriate injection timing, human AD-MSCs and UC-MSCs were injected on day 3 (early injection) or 7 (late injection) into mice with DSS-induced colitis, and the therapeutic effects were analyzed on days 11 and 21 after DSS induction. In the early injection group, both AD-MSCs and UC-MSCs significantly improved the DAI, colon length, and histological score compared with those in the control (Figs. 2 and 3). No differences in therapeutic effects between AD-MSCs and UC-MSCs were detected for DAI, colon length, and histological score.

In addition, real-time PCR analysis of inflammatory cytokines (*Il-6*, *Tnf-α*, *Il-17a*), anti-inflammatory cytokines (*Il-10*, transforming growth factor-beta [*Tgf-β*]), and vascular endothelial growth factor (*Vegf*) in colon tissues was performed. The results showed that on day 11, the mRNA levels of inflammatory cytokines *Il-6* and *Tnf* tended to decrease, although the difference was not significant, whereas on day 21, mRNA levels of inflammatory cytokines *Il-6*, *Tnf-α*, and *Il-17a* decreased significantly compared with those in the control group. On days 11 and 21, mRNA levels of *Il-10*, *Tgf-β*, and *Vegf* were not obviously changed, except for a decrease in *Il-10* mRNA on day 21 (Figs. 2D and 3D). Therefore, early timing of AD-MSC and UC-MSC injection ameliorated DSS-induced colitis in mice.

### Late timing of AD-MSC and UC-MSC injection did not ameliorate DSS-induced colitis

**FIG. 4.**
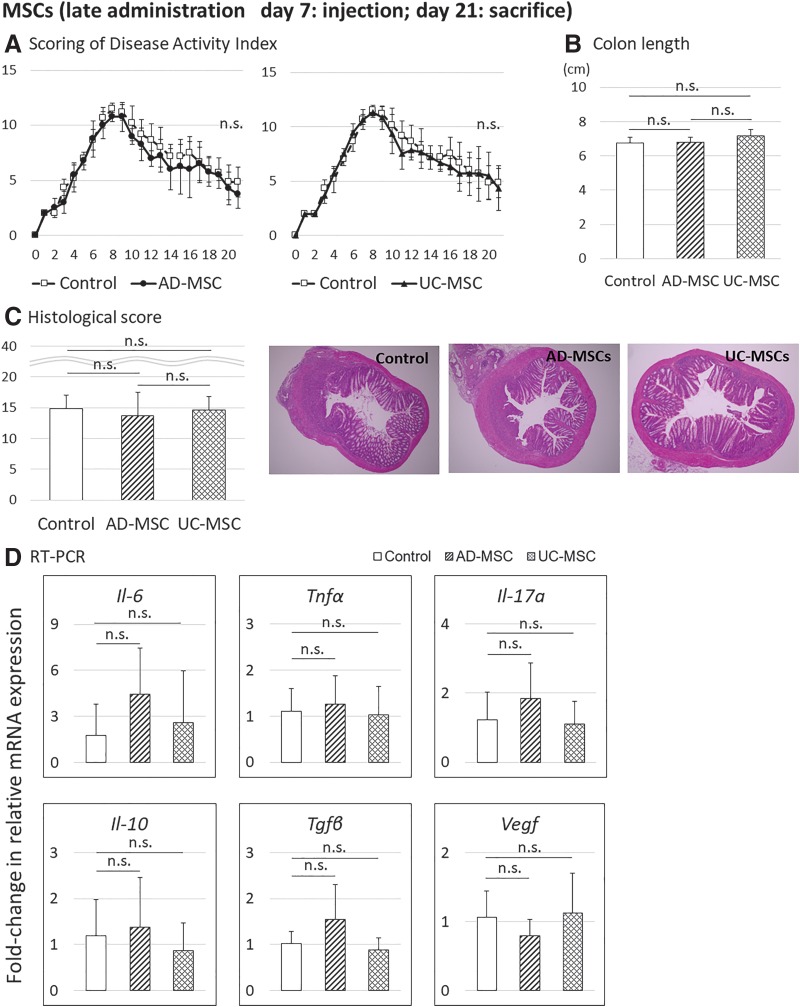
Evaluation of the therapeutic effects of AD-MSCs and UC-MSCs in a mouse model of DSS-induced colitis, after administration on day 7 and sacrifice on day 21. Data are presented as the means ± SDs (*n* = 16 mice: control, 6 mice; AD-MSCs, 4 mice; UC-MSCs, 6 mice) for each experiment. **(A)** DAI scores. **(B)** Colon length. **(C)** Histological scores. **(D)** Analysis of changes in mRNA expression compared with controls, as assessed by real-time PCR, in the middle colon. n.s. not significant.

Next, we evaluated the therapeutic effects of late injection of human AD-MSCs and UC-MSCs in mice with DSS-induced colitis. In contrast to the results of the early injection group, late injection did not show evidence of therapeutic effects, based on the DAI, colon length, and histological score on day 21 (Fig. 4). Real-time PCR analysis of inflammatory cytokines (*Il-6*, *TNF-α*, *Il-17a*), anti-inflammatory cytokines (*Il-10*, *Tgf-β*), and *Vegf* using colon tissues on day 21 revealed that there were no significant differences in these markers in the AD-MSC and UC-MSC groups. Therefore, the late timing of MSC injection did not ameliorate DSS-induced colitis, and proper timing is critical for alleviating inflammation in DSS-induced colitis (Fig. 4D).

### Supernatants from MSC cultures showed reduced therapeutic effects compared with MSC injection

**FIG. 5.**
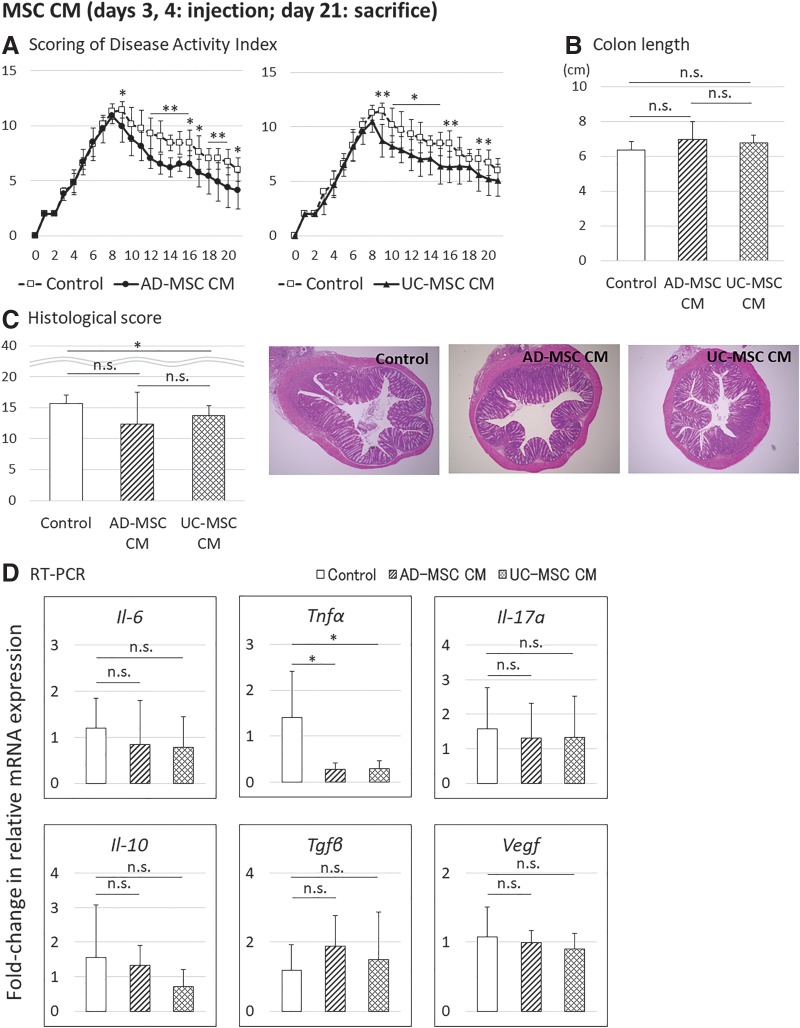
Evaluation of the therapeutic effects of AD-MSC CM and UC-MSC (UC-MSC CM) administered on days 3 and 4 after initiation of DSS treatment in mice. Data are presented as the means ± SDs (*n* = 27 mice: control, 7 mice; AD-MSC CM, 10 mice; UC-MSC CM, 10 mice) for each experiment. **(A)** DAI scores. **(B)** Colon length. **(C)** Histological scores. **(D)** Analysis of changes in mRNA expression in the middle colon compared with that in the untreated group. *p < 0.05; **p < 0.01.

We next evaluated the therapeutic effects of supernatants obtained from AD-MSC and UC-MSC cultures by comparing medium only without injection of cells. In the supernatant injection groups, the DAI, colon length, and histological score improved slightly compared with those of the control on day 21 (Fig. 5A–C). Real-time PCR analysis using the colon tissues on day 21 revealed that *Tnf-α* mRNA in the supernatant injection group decreased compared with that in the control medium injection group for both AD-MSCs and UC-MSCs (Fig. 5D). No differences in the therapeutic effects of AD-MSCs and UC-MSCs were detected in terms of the DAI, colon length, and histological score. Therefore, supernatants obtained from MSCs had some therapeutic effects, but those from MSC culture showed reduced therapeutic effects compared with injection of MSCs directly.

### RNA-seq analysis of colon tissues after MSC injection activated the anti-inflammatory reaction during very early timing

To identify the events occurring in the colon after AD-MSC and UC-MSC injection, we used NGS a day after cell injection (day 3: MSC injection; day 4: colons were obtained and analyzed). We then performed gene expression and pathway analyses using genes showing at least twofold changes in expression and having *p*-values of <0.05. We identified 451 mRNAs in the AD-MSC injection group, 893 mRNAs in the UC-MSC injection group, and 74 commonly expressed mRNAs (Supplementary Table S2). In these 74 mRNAs, we observed decrease in the B cell marker *Cd19* and T-helper cell type 17-related marker *Ccr6*. Pathway analysis using differentially expressed mRNAs revealed 12 pathways in the AD-MSC injection group and 26 pathways in the UC-MSC injection group (Supplementary Tables S3 and S4). The common pathways of both groups were nonodorant G protein-coupled receptors and complement and coagulation cascades.

**FIG. 6.**
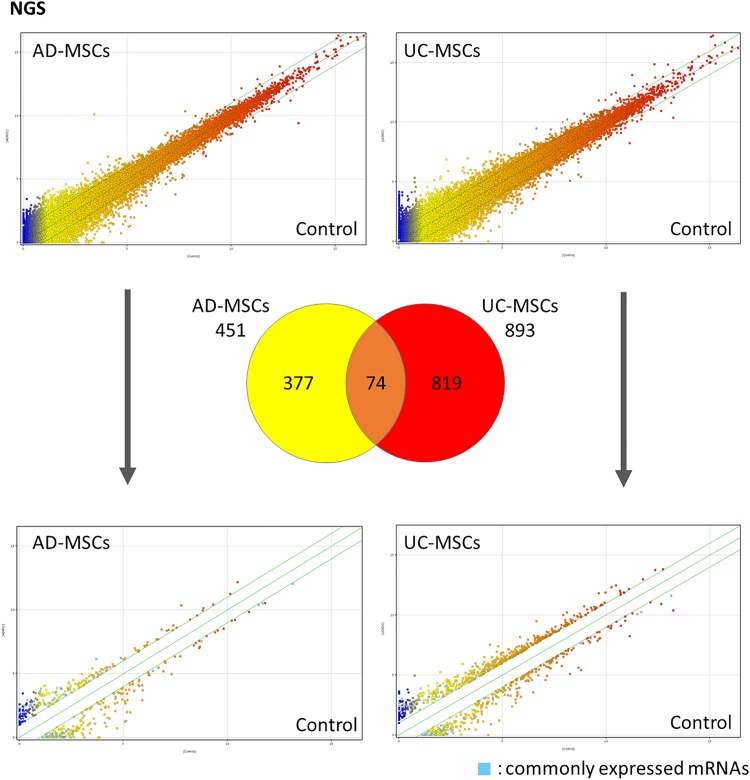
Evaluation of changes in mRNA expression in the colon after MSC administration using next-generation sequencing. Genes were considered differentially expressed if the fold change was <0.5 or >2.0 and the *p*-value was <0.05. Data were obtained from three mice for each group. MSC, mesenchymal stem cell.

Further analysis using differentially expressed mRNAs extracted only based on *p*-values of <0.05 yielded 1627 mRNAs in the AD-MSC injection group, 3064 mRNAs in the UC-MSC injection group, and 525 commonly expressed mRNAs. Pathway analysis using these mRNA revealed 50 pathways in the AD-MSC injection group and 89 pathways in the UC-MSC injection group. The commonly affected pathways for both groups were the TNF-α/nuclear factor (NF)-κB, T cell receptor, and epidermal growth factor receptor 1 (EGFR1) signaling pathways, suggesting that MSC injection affected the inflammatory response and fixation of epithelial cells after early timing of cell injection (Fig. 6). These data are registered with the Gene Expression Omnibus (GEO; accession No. GSE136397).

### Changes in mRNA expression in MSCs after serum addition demonstrated the anti-inflammatory effects of MSC therapy

**FIG. 7.**
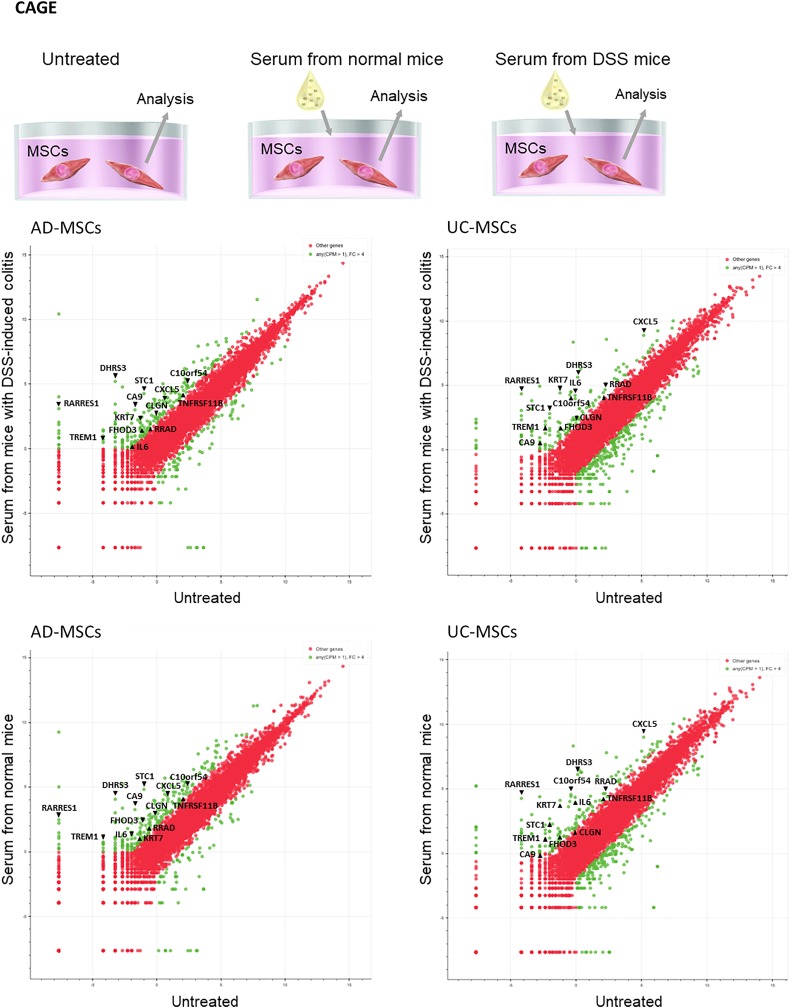
Factors from MSCs affecting inflammation, as analyzed using CAGE in AD-MSCs and UC-MSCs cultured without mouse serum, MSCs cultured with normal mouse serum, and MSCs cultured with serum from mice with DSS-induced colitis. The green dots show mRNAs with fourfold changes in expression. Data were obtained from three samples for each group. CAGE, cap analysis of gene expression.

To elucidate the anti-inflammatory factors of MSCs, CAGE analysis of MSCs treated with mouse serum was performed. Both AD-MSCs and UC-MSCs alleviated DSS-induced colitis; thus, we selected mRNAs showing at least fourfold increases in expression in MSCs cultured with serum derived from normal mice or from mice with DSS-induced colitis. As shown in the GEO (accession No. GSE137173), 215 and 73 mRNAs (serum derived from normal mice versus untreated) and 206 and 93 mRNAs (serum derived from mice with DSS-induced colitis versus untreated) were upregulated and downregulated, respectively, in AD-MSCs, whereas 142 and 206 mRNAs (serum derived from normal mice versus untreated) and 130 and 252 mRNAs (serum derived from mice with DSS-induced colitis versus untreated) were upregulated and downregulated, respectively, in UC-MSCs. The commonly upregulated mRNAs included chromosome 10 open reading frame 54 (*C10orf54*), carbonic anhydrase IX (*CA9*), calmegin (*CLGN*), chemokine ligand 5 (*CXCL5*), dehydrogenase/reductase (SDR family) member 3 (*DHRS3*), formin homology 2 domain containing 3 (*FHOD3*), *IL-6*, keratin 7 (*KRT7*), retinoic acid receptor responder protein 1 (*RARRES1*), Ras-related associated with diabetes (*RRAD*), stanniocalcin-1 (*STC1*), TNF receptor superfamily member 11b (*TNFRSF11B*), and triggering receptor expressed on myeloid cells 1 (*TERM1*). Most of these factors have been reported to affect inflammation (Fig. 7).

### MSC therapy maintained levels of Bacteroidetes and Firmicutes in the intestinal flora

**FIG. 8.**
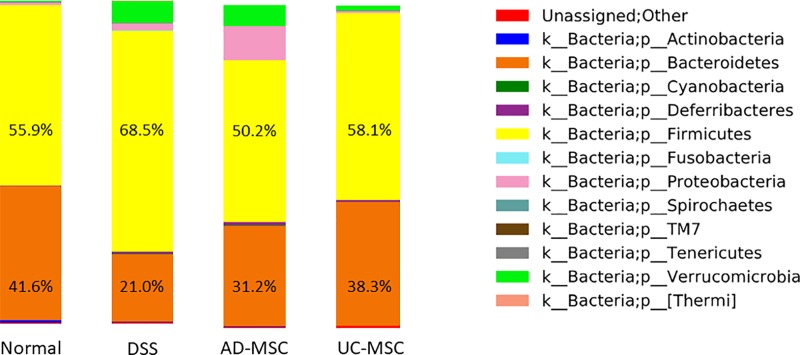
Evaluation of changes in intestinal flora induced by AD-MSCs and UC-MSCs. The frequencies of Bacteroidetes and Firmicutes in each group are shown as bars. Data were obtained from three mice for each group.

The intestinal flora affects disease progression. Therefore, we evaluated the intestinal flora on day 11 after the start of the experiment. Our results showed that the frequencies of Bacteroidetes and Firmicutes, which are known to decrease in number in patients with IBD, tended to be maintained in mice with DSS-induced colitis injected with AD-MSCs (Bacteroidetes: 31.2% ± 18.5%, Firmicutes: 49.2% ± 33.5%) or with UC-MSCs (Bacteroidetes: 38.1% ± 13.3%, Firmicutes: 58.3% ± 13.4%) compared with those in mice with DSS-induced colitis without cell administration (Bacteroidetes: 21.2% ± 7.8%, Firmicutes: 68.0% ± 10.0%). In the normal control group, the percentages of Bacteroidetes and Firmicutes were 41.5% ± 3.5% and 56.0% ± 3.6%, respectively (Fig. 8). These results showed that Bacteroidetes and Firmicutes in the intestinal flora tended to be maintained by MSC therapy.

## Discussion

In this study, we showed that the therapeutic effects of AD-MSCs and UC-MSCs were similar; however, early cell injection after DSS-induced colitis was more effective than late cell injection. Furthermore, the supernatants of MSC cultures were partially effective, although injection of cells directly had stronger anti-inflammatory effects. During the analysis, we identified potential signaling pathways by NGS using colon tissues on day 1 after cell administration; the results revealed the effects of MSCs on changes in anti-inflammatory factors and the intestinal flora.

Our findings confirmed previous results showing that human MSCs have therapeutic effects in animal models of experimental colitis.^15,16^ These results have demonstrated the low antigenicity of MSCs, which is essential for clinical applications. Autologous and allogeneic MSCs can be safely used for many diseases, including disorders of the neurons, liver, muscles, and gastrointestinal tract, and can be obtained from medical wastes. In particular, allogeneic cells can be used to prepare the cells before disease onset and can facilitate rapid administration, allowing MSC therapy to be used for the treatment of various diseases.

Kawata et al. showed that early injection was more effective using AD-MSCs. Our results are in agreement with this finding, and we showed that this timing effect was universal in both AD-MSCs and UC-MSCs. Our results were also consistent with a report showing that MSCs exhibit increased therapeutic effects during the inflammatory phase than during the reparative and remodeling phases.^17^ In addition, dexamethasone weakens the therapeutic effects of MSCs in a mouse model of CCl_4_-induced liver injury. The concept that appropriate cell injection timing and host inflammatory conditions induce the maximum therapeutic effect is important when MSCs are used for the treatment of inflammatory diseases.^18^

In this study, the partial effect of cell culture supernatants suggested that trophic factors of MSCs had therapeutic effects. Moreover, the therapeutic effects of the supernatant were not strong compared with those reported previously; however, we believe that trophic factors are important for treatment outcomes. Watanabe et al. reported that supernatants of MSCs are effective for DSS-induced colitis treatment in mice; however, we obtained different results.^19^ Notably, Lee et al. reported that after MSCs were infused into mice, most of the cells were trapped as embolizations in the lungs, and 99% of cells were cleared from circulation within 5 min.^20^ We observed a similar result; when we injected DsRed-positive MSCs via the tail vein into a mouse model of CCl4-induced liver cirrhosis and observed lung, liver, and spleen tissues by two-photon microscopy, most MSCs were trapped in the lungs, and very few cells migrated to the liver.^21^ These cell behaviors strongly suggest that the effects of MSCs are indirect.

Accordingly, we assumed that in our experiment, most of the MSCs did not migrate to the colon and that humoral factors caused the observed therapeutic effects. Recently, Katsuda et al. reported that exosomes derived from MSCs could have therapeutic effects similar to those of MSC injection.^22^ Thus, further studies are needed to identify the specific factors or conditions needed for MSCs to exert their therapeutic effects.

Accordingly, in this study, we analyzed changes in gene expression induced by MSCs and showed that *C10orf54*, *CA9*, *CLGN*, *CXCL5*, *DHRS3*, *FHOD3*, *IL-6*, *KRT7*, *RARRES1*, *RRAD*, *STC1*, *TNFRSF11B*, and *TERM1* mRNAs were commonly upregulated when MSCs were stimulated with serum. Among these, we selected *C10orf54*, *STC1*, and *TNFRSF11B* for further evaluation.

C10orf54, which encodes V-domain Ig suppressor of T cell activation (VISTA), is a known negative regulator of T cells. VISTA, which was first reported by Wang et al., has been shown to be highly regulated on myeloid antigen-presenting cells and T cells.^23^ This molecule is related to autoimmunity and immune surveillance in cancer, and its protective roles in anticancer immunity have been of particular interest. Le Mercier et al. reported that VISTA acts as a negative checkpoint regulator to suppress T cell activation and induce Foxp3 expression and is highly expressed within the tumor microenvironment.^24^ In addition, Ceeraz et al. reported that in VISTA-deficient mice, surface expression of the C5a receptor was reduced on monocytes, neutrophils, and cultured macrophages, suggesting that VISTA supports optimal responses to C5a and modulates macrophage responses to immune complexes.^25^

STC-1 is a multifunctional glycoprotein with antioxidant and anti-inflammatory properties. Mohammadipoor et al. reported that the beneficial effects of STC-1 may be attributed to CD14 suppression on recruited monocytes and macrophages, limiting their inflammatory responses.^26^ Moreover, Oh et al. reported that recombinant STC-1 reproduces the effects of MSCs on the inhibition of NLRP3 inflammasome activation and reactive oxygen species production in macrophages and concluded that human MSCs inhibit NLRP3 inflammasome activation in macrophages primarily by secreting STC-1 in response to activated macrophages and thus decreasing mitochondrial loss.^27^ Similarly, Ono et al. reported that STC-1 from MSCs tends to abolish inappropriate epithelial–mesenchymal relationships in pulmonary fibrosis.^28^

TNFRSF11B, which is also known as osteoprotegerin (OPG), was first discovered as a novel secreted TNF receptor-related protein and has been shown to play roles in the regulation of bone density and as a decoy receptor for receptor activator of nuclear factor kappa-B ligand (RANKL).^29^ RANKL secreted by mesenchymal cells in the subepithelium interacts with the gut epithelium to control CCL20 expression and microfold (M) cell differentiation. The deletion of RANKL impairs M cell-dependent antigen sampling and B cell/dendritic cell interactions in the subepithelial dome, resulting in reduced IgA production and decreased microbial diversity.^30^

In our study, we found that flora from MSC-injected mice was similar to that in mice without DSS-induced colitis. Although we did not evaluate the effects of OPG in this model, our findings suggest that OPG may have contributed to these results.

In our tissue analysis, we found that the TNF-α/NF-κB, T cell receptor, and EGFR1 signaling pathways were altered. These results were consistent with our real-time PCR analysis, showing that *TNF-α* and *IL-17α* were downregulated after cell injection. Decreased TNF-α signaling immediately after cell injection is important for mediating the multiple effects of MSC administration. Moreover, NF-κB signaling can regulate subsequent inflammatory signals, including IL-1 and IL-6, and can exert antiapoptotic effects. Together, alterations in these two pathways can contribute to the observed therapeutic effects.

In addition, our previous analysis revealed that MSCs show reduced expansion and activation of T cells *in vitro* and induction of regulatory T cells *in vivo.*^17^ Given that this NGS analysis was performed the day after cell injection and that most MSCs were trapped in the lungs at this time point, we believe that humoral factors, including exosomes, should have been able to affect the colon tissue.

## Conclusion

Our results showed that MSCs had various direct and indirect effects on DSS-induced colitis. Further studies are needed to assess the optimal conditions for donors, cell origins, and preconditioning regimens. However, our findings supported that for some patients with IBDs, MSCs or substances released from MSCs may act as novel tools for controlling inflammation.

## Supplementary Material

Supplemental data

Supplemental data

## Abbreviations Used

AD-MSCs: adipose tissue-derived mesenchymal stem cells
*C10orf54*: chromosome 10 open reading frame 54
*CA9*: carbonic anhydrase IX
CAGE: cap analysis of gene expression
CD: Crohn's disease
*CLGN*: calmegin
CM: conditioned medium
*CXCL5*: chemokine ligand 5
DAI: disease activity index
*DHRS3*: dehydrogenase/reductase (SDR family) member 3
DSS: dextran sulfate sodium
EGFR1: epidermal growth factor receptor 1
*FHOD3*: formin homology 2 domain containing 3
GEO: Gene Expression Omnibus
HLA: human leukocyte antigen
IBDs: inflammatory bowel diseases
IL: interleukin
*KRT7*: keratin 7
M: microfold
mRNA: messenger RNA
MSCs: mesenchymal stem cells
NF: nuclear factor
NGS: next-generation sequencing
OPG: osteoprotegerin
PCR: polymerase chain reaction
RANKL: receptor activator of nuclear factor kappa-B ligand
*RARRES1*: retinoic acid receptor responder protein 1
RNA-seq: RNA-sequencing
*RRAD*: Ras-related associated with diabetes
*STC1*: stanniocalcin-1
*TERM1*: triggering receptor expressed on myeloid cells 1
*Tgf-β*: transforming growth factor-beta
TNF: tumor necrosis factor
*TNFRSF11B*: TNF receptor superfamily member 11b
UC: ulcerative colitis
UC-MSCs: umbilical cord tissue-derived mesenchymal stem cells
*Vegf*: vascular endothelial growth factor
VISTA: V-domain Ig suppressor of T-cell activation
