# Supplementary material for: Effects of Human Adipose Tissue-Derived and Umbilical Cord Tissue-Derived Mesenchymal Stem Cells in a Dextran Sulfate Sodium-Induced Mouse Model
Source: Biores Open Access. 2019 Nov 11;8(1):185–99. doi: 10.1089/biores.2019.0022 (PMC6844129; doi:10.1089/biores.2019.0022)
Supplement: Supplemental data [file Suppl_TableS1-S3.pdf]

Supplementary Data

Supplementary Table S1. List of Primers Used for Real-Time Polymerase Chain Reaction

Primer	Catalog No.	Species	Company
Gapdh	QT01658692	Mouse	Qiagen
Il-6	QT00098875	Mouse	Qiagen
Tnfα (Tnf)	QT00104006	Mouse	Qiagen
Il-17a	QT00103278	Mouse	Qiagen
Il-10	QT00106169	Mouse	Qiagen
Tgfβ	QT00145250	Mouse	Qiagen
Vegf	QT00160769	Mouse	Qiagen

IL, interleukin; Tnf, tumor necrosis factor; Vegf, vascular endothelial growth factor.

Supplementary Table S2. List of 74 Commonly Expressed Messenger RNAs (Upregulated or Downregulated by at Least Twofold, with $p < 0.05$)

	Gene ID	Gene symbol	AD-MSC	UC-MSC
1	ENSMUSG00000027015	*Cybrd1*	Up	Up
2	ENSMUSG00000086328	*2700033N17Rik*	Up	Up
3	ENSMUSG00000086605	*Gm14290*	Up	Up
4	ENSMUSG00000069074	*Gm10258*	Up	Down
5	ENSMUSG00000074637	*Sox2*	Up	Up
6	ENSMUSG00000051279	*Gdf6*	Up	Up
7	ENSMUSG00000094595	*Fsbp*	Up	Up
8	ENSMUSG00000066000	*2610305D13Rik*	Up	Up
9	ENSMUSG00000028940	*Hes2*	Up	Up
10	ENSMUSG00000029490	*Mfsd7a*	Up	Up
11	ENSMUSG00000019577	*Pdk4*	Up	Up
12	ENSMUSG00000087389	*Gm15592*	Up	Down
13	ENSMUSG00000074355	*Gm10676*	Up	Up
14	ENSMUSG00000030474	*Siglece*	Up	Up
15	ENSMUSG00000097388	*RP24-98O8.1*	Up	Up
16	ENSMUSG00000081431	*Gm15483*	Up	Up
17	ENSMUSG00000090334	*Gm17149*	Up	Up
18	ENSMUSG00000032034	*Kcnj5*	Up	Up
19	ENSMUSG00000005131	*4930550C14Rik*	Up	Up
20	ENSMUSG00000085687	*Gm16153*	Up	Up
21	ENSMUSG00000047692	*4930533K18Rik*	Up	Up
22	ENSMUSG00000090665	*Gad1-ps*	Up	Up
23	ENSMUSG00000018238	*Gdf9*	Up	Up
24	ENSMUSG00000001508	*Sgca*	Up	Up
25	ENSMUSG00000050538	*B230217C12Rik*	Up	Up
26	ENSMUSG00000025375	*Aatk*	Up	Up
27	ENSMUSG00000037169	*Mycn*	Up	Up
28	ENSMUSG00000079143	*Gm11052*	Up	Up
29	ENSMUSG00000084691	*SNORD113*	Up	Up
30	ENSMUSG00000096464	*AC090887.7*	Up	Up
31	ENSMUSG00000041707	*1810011H11Rik*	Up	Up
32	ENSMUSG00000038984	*Tspyl5*	Up	Up
33	ENSMUSG00000054409	*Tmem74*	Up	Up
34	ENSMUSG00000089525	*7SK*	Up	Up
35	ENSMUSG00000089952	*4933413C19Rik*	Up	Up
36	ENSMUSG00000033717	*Adra2a*	Up	Up
37	ENSMUSG00000090682	*Gm3852*	Down	Down
38	ENSMUSG00000090323	*Gm5263*	Down	Down
39	ENSMUSG00000052403	*Gm13520*	Down	Down
40	ENSMUSG00000082455	*Gm13665*	Down	Down
41	ENSMUSG00000075174	*Olfr1087*	Down	Down
42	ENSMUSG00000074771	*Ankrd5*	Down	Down
43	ENSMUSG00000062124	*Defb45*	Down	Down
44	ENSMUSG00000046688	*Tifa*	Down	Down
45	ENSMUSG00000037994	*Nhedc2*	Down	Down
46	ENSMUSG00000082368	*Gm11225*	Down	Down
47	ENSMUSG00000084162	*Gm11251*	Down	Down
48	ENSMUSG00000097554	*AC092404.1*	Down	Down
49	ENSMUSG00000083840	*Gm15459*	Down	Down
50	ENSMUSG00000046000	*Naa11*	Down	Down
51	ENSMUSG00000092956	*AC044807.1*	Down	Down
52	ENSMUSG00000072677	*Gm10396*	Down	Down
53	ENSMUSG00000086433	*1700006D18Rik*	Down	Down
54	ENSMUSG00000030724	*Cd19*	Down	Down
55	ENSMUSG00000083396	*Gm15542*	Down	Down
56	ENSMUSG00000046441	*Ftsjd1*	Down	Down
57	ENSMUSG00000015656	*Hspa8*	Down	Down
58	ENSMUSG00000047990	*C2cd4a*	Down	Down
59	ENSMUSG00000035606	*Ky*	Down	Down
60	ENSMUSG00000037542	*Aldh8a1*	Down	Down
61	ENSMUSG00000089839	*Rps19-ps11*	Down	Down
62	ENSMUSG00000083748	*Gm11662*	Down	Down
63	ENSMUSG00000083356	*Gm11708*	Down	Down
64	ENSMUSG00000095265	*AC079644.1*	Down	Down
65	ENSMUSG00000091577	*Gm6211*	Down	Down
66	ENSMUSG00000082662	*Gm15516*	Down	Down
67	ENSMUSG00000075427	*Olfr288*	Down	Down
68	ENSMUSG00000040899	*Ccr6*	Down	Down
69	ENSMUSG00000052270	*Fpr2*	Down	Down
70	ENSMUSG00000024402	*Lta*	Down	Down
71	ENSMUSG00000055760	*Gemin6*	Down	Down
72	ENSMUSG00000032845	*Alpk2*	Down	Down
73	ENSMUSG00000082461	*Gm8844*	Down	Down
74	ENSMUSG00000045694	*Gm4997*	Down	Down

(*continued*)

Supplementary Table S2. (continued)

AD-MSC, adipose tissue-derived mesenchymal stem cell; UC-MSC, umbilical cord tissue-derived mesenchymal stem cell.

Supplementary Table S3. List of 12 Pathways in the Adipose Tissue-Derived Mesenchymal Stem Cell Injection Group (Pathway Analysis Using Messenger RNAs That Were Upregulated or Downregulated by at Least Twofold, with $p < 0.05$)

	Pathway	*p*
1	Mm_Myometrial_Relaxation_and_Contraction_Pathways_WP385_72108	0.0395067
2	Mm_Non-odorant_GPCRs_WP1396_69993	0.0047186
3	Mm_PPAR_signaling_pathway_WP2316_69143	0.0145176
4	Mm_Peptide_GPCRs_WP234_69827	0.0487054
5	Mm_GPCRs,_Class_A_Rhodopsin-like_WP189_71758	0.0175097
6	Mm_Steroid_Biosynthesis_WP55_71743	0.0085364
7	Mm_Calcium_Regulation_in_the_Cardiac_Cell_WP553_73390	0.0021501
8	Mm_Complement_and_Coagulation_Cascades_WP449_71733	0.0059489
9	Mm_Glucocorticoid_&_Mineralcorticoid_Metabolism_WP495_71740	0.0085364
10	Mm_B_Cell_Receptor_Signaling_Pathway_WP274_67072	0.0109583
11	Mm_Dopminergic_Neurogenesis_WP1498_60839	0.0489400
12	Mm_Eicosanoid_Synthesis_WP318_71719	0.0209433
