# Supplementary material for: Effects of Human Adipose Tissue-Derived and Umbilical Cord Tissue-Derived Mesenchymal Stem Cells in a Dextran Sulfate Sodium-Induced Mouse Model
Source: Biores Open Access. 2019 Nov 11;8(1):185–99. doi: 10.1089/biores.2019.0022 (PMC6844129; doi:10.1089/biores.2019.0022)
Supplement: Supplemental data [file Suppl_TableS4.pdf]

Supplementary Table S4. List of 26 Pathways in the Umbilical Cord Tissue-Derived Mesenchymal Stem Cell Injection Group (Pathway Analysis Using Messenger RNAs That Were Upregulated or Downregulated by at Least Twofold, with $p < 0.05$)

	Pathway	*p*
1	Mm_Focal_Adhesion_WP85_69966	0.0000001
2	Mm_Focal_Adhesion-PI3K-Akt-mTOR-signaling_pathway_WP2841_77383	0.0000005
3	Mm_Spinal_Cord_Injury_WP2432_71041	0.0000006
4	Mm_XPodNet_-_protein-protein_interactions_in_the_podocyte_expanded_by_STRING_WP2309_72004	0.0000082
5	Mm_Complement_and_Coagulation_Cascades_WP449_71733	0.0000120
6	Mm_Endochondral_Ossification_WP1270_72216	0.0000137
7	Mm_Non-odorant_GPCRs_WP1396_69993	0.0000199
8	Mm_TGF_Beta_Signaling_Pathway_WP113_69818	0.0000267
9	Mm_Complement_Activation,_Classical_Pathway_WP200_72061	0.0000343
10	Mm_Matrix_Metalloproteinases_WP441_69114	0.0000489
11	Mm_Inflammatory_Response_Pathway_WP458_71731	0.0000599
12	Mm_Peptide_GPCRs_WP234_69827	0.0002093
13	Mm_GPCRs,_Class_A_Rhodopsin-like_WP189_71758	0.0002766
14	Mm_Urea_cycle_and_metabolism_of_amino_groups_WP426_72149	0.0010778
15	Mm_Cell_cycle_WP190_71755	0.0041164
16	Mm_PodNet-_protein-protein_interactions_in_the_podocyte_WP2310_72005	0.0079890
17	Mm_SIDS_Susceptibility_Pathways_WP1266_69139	0.0141451
18	Mm_Fatty_Acid_Biosynthesis_WP336_71737	0.0141904
19	Mm_Splicing_factor_NOVA_regulated_synpatic_proteins_WP1983_71717	0.0165957
20	Mm_Oxidative_Stress_WP412_69190	0.0180174
21	Mm_Metapathway_biotransformation_WP1251_69747	0.0198458
22	Mm_Osteoblast_WP238_72001	0.0218098
23	Mm_Neural_Crest_Differentiation_WP2074_69080	0.0267661
24	Mm_Prostaglandin_Synthesis_and_Regulation_WP374_69204	0.0354852
25	Mm_Estrogen_metabolism_WP1264_69069	0.0360929
26	Mm_Keap1-Nrf2_WP1245_71125	0.0414660
